# Morphological Characteristics of Grapevine Cultivars and Closed Contour Analysis with Elliptic Fourier Descriptors

**DOI:** 10.3390/plants10071350

**Published:** 2021-07-01

**Authors:** Muhammed Kupe, Bahadır Sayıncı, Bunyamin Demir, Sezai Ercisli, Mojmir Baron, Jiri Sochor

**Affiliations:** 1 Department of Horticulture, Faculty of Agriculture, Atatürk University, Erzurum 25240, Turkey; muhammed.kupe@atauni.edu.tr (M.K.); sercisli@atauni.edu.tr (S.E.); 2 Department of Mechanical Engineering, Faculty of Engineering, Mersin University, Mersin 33340, Turkey; bsayinci@mersin.edu.tr; 3 Department of Viticulture and Enology, Faculty of Horticulture, Mendel University in Brno, Valticka 337, 691 44 Lednice, Czech Republic; mojmirbaron@seznam.cz (M.B.); jiri.sochor@mendelu.cz (J.S.)

**Keywords:** morphological analysis, projected area, dimensional analysis, contour analysis, sphericity

## Abstract

Morphology is the most visible and distinct character of plant organs and is accepted as one of the most important tools for plant biologists, plant breeders and growers. A number of methods based on plant morphology are applied to discriminate in particular close cultivars. In this study, image processing analysis was used on 20 grape cultivars (“Amasya beyazı“, “Antep karası“, “Bahçeli karası”, “Çavuş“, “Cevşen“, “Crimson“, “Dimrit“, “Erenköy beyazı“, “Hafızali“, “Karaşabi“, “Kırmızı“, “İzabella (Isabella) “, “Morşabi“, “Müşgüle“, “Nuniya“, “Royal“, “Sultani çekirdeksiz (Sultanina)“, “Yalova incisi“, “Yerli beyazv“, “Yuvarlak çekirdeksiz“) to classify them. According to image processing analysis, the longest and the greatest projected area values were observed in “Antep karası“ cultivar. The “Sultani çekirdeksiz“ cultivar had the least geometric mean diameter. The greatest sphericity ratios were observed in “Yerli beyaz“, “Erenköy beyazı“ and “Amasya beyazı“ cultivars. According to principal component analysis, dimensional attributes were identified as the most significant source of variation discriminant grape cultivars from each other. Morphological differences between the cultivars were explained by sphericity and elongation variables. According to elliptic Fourier analysis (EFA) results, grape morphology largely looks like ellipse and sphere. However, there are some cultivars that look similar to a water drop. The cultivars with similar morphology were identified by a pair-wise comparison test conducted with the use of linear discriminant analysis, and they were presented in a scatter plot. According to cluster analysis, present grape cultivars were classified into seven sub-groups, which indicated great diversity.

## 1. Introduction

Grape is one of the oldest horticultural crops. It is also one of the most cultivated horticultural plants, along with apple, citrus and banana. Total world grape production in 2018 was 79,000,000 metric tons, up by 6.5% from 74,000,000 tons in 2017. China was the largest producer of grapes, with 13,397,000 tons of production, followed, respectively, by Italy with 8,514,000 tons, the USA with 6,891,000 tons, Spain with 6,673,00 tons, France with 6,198,000 tons and Turkey with 4,000,000 tons of production [1].

Worldwide, about 57% of grapes are used to make wine, 36% are consumed as fresh table grapes and 7% as dried grapes [2].

Common grapevines, *Vitis vinifera* L. are widely distributed, mainly in the temperate and subtropical regions in the world, including Mediterranean countries, Central and Southern Europe, to southwestern Asia. It is estimated that 10000 known grapevine varieties are distributed in grape growing areas throughout the world, and around 13 varieties dominate world production and cover more than one-third of the world’s vineyard area [3].

The neighboring regions of the Caucasian area, including Turkey, have a long history of viticulture and possess a great diversity of local grape cultivars into different products. In Turkey, a large number of wild grapevines (*Vitis vinifera* L. subsp. *sylvestris*) and local cultivars are available [4]. The processes of cultivar selection and breeding started with wild grapes *Vitis vinifera* L. ssp. *sylvestris* Gmel. and included multiple introgression events from the wild to domestication [5], and it was initiated 8000 years ago in the South Caucasian area [6].

Previous archeological, palaeobotanical and historical studies confirm that grapevines were spread and cultivated for a long time in the Anatolia region in Turkey and have been an important part of civilizations established there [4,7]. In Anatolia, viticulture and wine-making have been developed for centuries by using local quite diverse grape cultivars. Those cultivars have been used for several purposes, such as for fresh consumption, drying, wine and spirit production and even for decorative and ornamental purposes [5,6]. During the last 3 decades, significant progress was achieved in the viticulture of Anatolia, and vineyards were diversified with foreign worldwide famous cultivars.

Turkey has more than 1500 national grape cultivars, of which 800 are genetically different. There are around 30 outstanding wine grape cultivars among them. The most suitable local Turkish grape cultivars for making wine are Bogazkere, Okuzgozu, Emir and Papazkarasi [7,8].

Local grape cultivars are mainly grown in old vineyards located in ancient settlements and homesteads. There are also different growing systems, including suspended and creeping cultivations. The local grape cultivars differ from each other by their morphological characteristics and sizes of the bunches and berries; phenology; time of harvest; productivity and quality indices [9,10,11]. Local grape cultivars are essential to sustain crop diversity and can also be essential for food, nutrition and economic security of many people-particularly smallholder farmers and farming communities in rural and marginal areas. The diversity in local grapes can provide assurance against crop failure and offer special materials for traditional local cuisines and specific dietary requirements. Furthermore, these diverse grape cultivars are an important source of locally adapted genes for the improvement of the new grape cultivars [12,13].

Turkey’s natural and ecological endowments are favorable for producing grapes. The grape industry could be an important part of agriculture from the point of view of employment and value creation. Due to its role in tourism and its opportunities for export, the industry can be regarded as one of the industries to be strategically developed. In addition, many regions of Turkey are rich in valuable local grapevine varieties which have not been explored and fully characterized yet [14].

In each grape growing country, there were numerous local varieties that contribute to world grapevine diversity [15,16]. Within a grape variety, significant clonal variability was evident [17,18]. Thus, the definition and the identification of varieties are of considerable scientific and practical importance in modern viticulture and ampelography [19,20,21,22].

Grape berries belonging to different cultivars show considerable diversity in berry morphology, in particular for berry size, color and shape. Berry size, color and shape attributes are of primary importance in the perceived quality and overall acceptability to consumer preference [10,11]. Correct cultivar identification in grapes is important to grape growers, regulatory authorities and winemakers. More recently, with the advertisement on grape berry composition, an increasing interest in new grapevine plantations has occurred, and there is a need to ensure trueness to the type of grape-planting material. Mistakes on variety trueness may result in significant financial costs not only for growers but also related industry [23]. For a long time, traditional grapevine variety identification was made by visual inspection of the grapevine that is known as ampelography. However, the use of ampelography in variety identification in grapevine varieties does not give an exact result, and some variability in descriptor definition may occur due to environmental conditions, cultural practices and genetic variations. For example, the same grape variety shows size, shape and color variability on berries and bunches in different environments. Health status, including diseases, can make classification more complex [24,25,26]. Ampelographic descriptions for a variety vary slightly according to the interpretation of the observer as well. In addition, it is important to establish relativity with the descriptors used, particularly in trying to distinguish between similar varieties [9,10,11]. In general, the berry shape index was used for descriptors in grapevine cultivars, but fruit shape is a three-dimensional characteristic and must be defined using pleiotropic explanatory variables rather than a simple/single index [27].

Recently, some sophisticated methods, including multivariate analysis [28], artificial neural network [29], DNA marker technologies including SSR and SNP [30,31] and Elliptic Fourier analysis [32], have been using in grapevine for ampelographic data processing and variety trueness. All these methods could be efficiently used to determine the differences or synonymy of grapevine genotypes.

Elliptic Fourier analysis (EFA) is gained more importance with the improvements of computer performance along with decreases in the cost of digital imaging hardware and software more recently. Thus the method widely contributed digital image processing applications for agriculturally relevant morphological analyses in different crop species [27,33,34,35]. These studies indicated that EFA provided an excellent tool for shape discrimination of several agricultural products. However, studies on EFA on grapevine cultivars are very limited in the literature. The main approach of this method is better defining the complex shape of fruits, etc. The method requires a set of coordinate values or descriptors obtained in a Fourier analysis [27]. This method determines the overall shape based on image data by first transforming coordinate information regarding the image contours into EFDs (Elliptic Fourier Descriptors), which are then summarized by a PCA (Principal Component Analysis). Analyses based on EFDs and PCA have been completed using the SHAPE program [36].

This study was conducted to investigate the size and shape features describing the physical attributes of 20 grapevine cultivars and to reveal shape distinctions with Elliptic Fourier descriptors modeling the closed contour of the cultivars.

## 2. Materials and Methods

### 2.1. Locations of Grape Cultivars

The study was conducted at the commercial farm Kemerhisar, Nigde province in middle Turkey during 2020. The geographical coordinates of the experiment location were 37.8318° N latitude and 34.6001° E longitude, and 1120 m elevation above sea level. All 20 grape cultivars were harvested from a 40-year-old wire-trained vineyard on the date of 12.09.2020. On the same day of harvest, samples were transported in cold-chain to Advanced Technology Research and Implementation Center of Mersin University. Daily irrigations from May to September and other management practices (herbicide and fertilizer applications and pruning) were conducted according to the farm manager’s criteria. Herbicides were periodically applied between rows to control weeds. Vines were pruned with shears in February each year. The berry skin color of grapevine cultivars is given in Table 1.

### 2.2. Imaging System and Sampling

**Figure 1:**
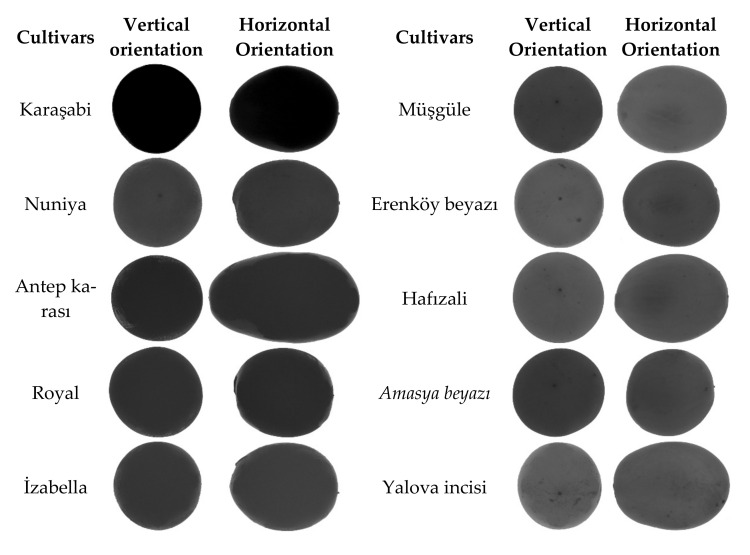

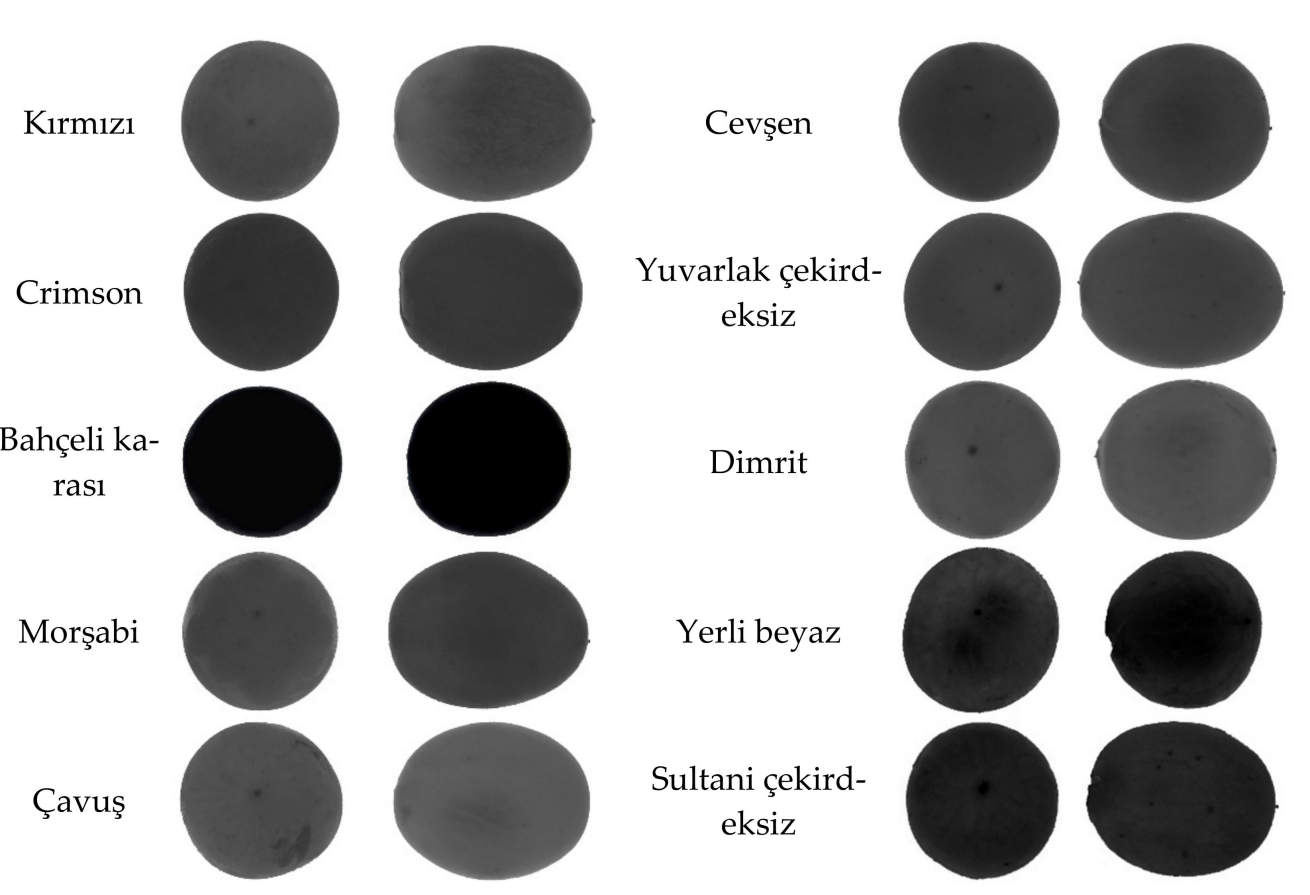
Grapevine cultivars.

**Figure 2:**
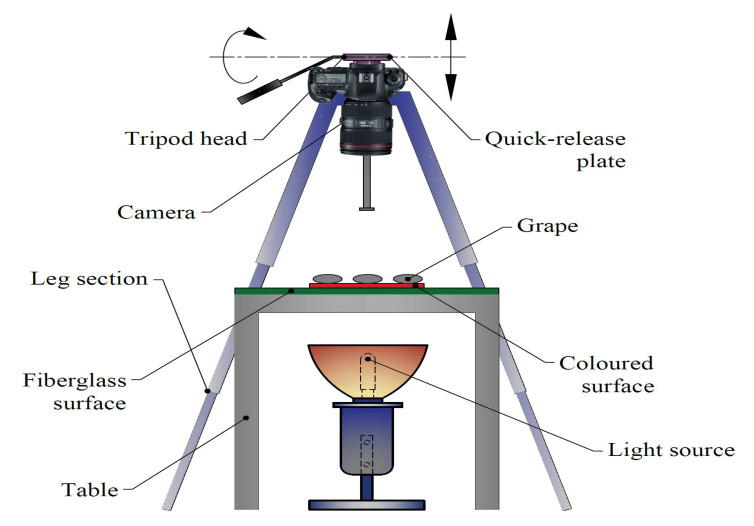
Image acquisition system.

Twenty grape cultivars used in present experiments are presented in Figure 1. For analyses, 40 berry samples were randomly selected from each cultivar. Berry samples were placed on white fiberglass plate in 4 × 5 matrix array in 2 groups. Cylinder-formed plastic supports were used to fixate samples in both horizontal and vertical orientation. Grape cultivars were imaged with the use of Nikon D90 model digital camera and image files with *.tiff extension were recorded. Imaging system is presented in Figure 2. Artificial lighting was provided beneath the fiberglass plate to clarify the contours of berry samples [37]. Transparent surfaces were used to provide a contrast between plate and berry color. Digital camera was mounted and fixed on a tripod. Imaging was performed 56 cm above the samples. A shutter release cord was used to prevent vibrations while taking the images. Grape samples were imaged at both horizontal and vertical orientations. A millimetric ruler was placed by the grape samples to convert pixel units into millimeters.

### 2.3. Morphology and Dimensional Attributes

**Figure 3:**
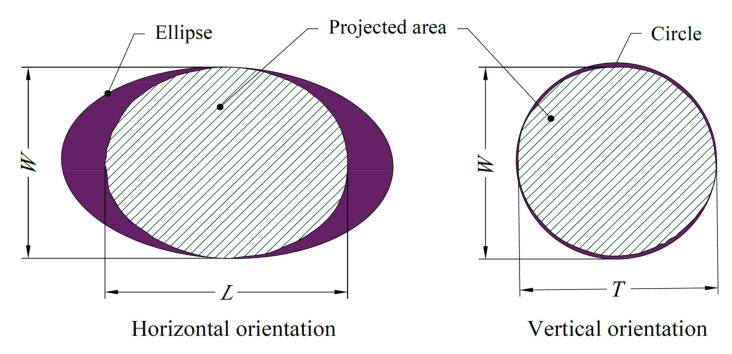
Size and area measurements of the grape cultivars.

SigmaScan^®^Pro 5.0 software was used to determine morphology and dimensional attributes of the grape cultivars. For image processing, 0–255 threshold range was applied to monochrome images and dimension analysis was automatically performed. Calibration was made over the ruler to convert pixel units into millimeters. With the present analyses, length (L, mm), width (W, mm), thickness (T, mm), projection area (PA, mm^2^), equivalent diameter (ED, mm), perimeter (P, mm) and circularity (C) were automatically measured. Dimensional and area measures are presented in Figure 3 and equations used in calculations are provided in Table 2.

### 2.4. Elliptic Fourier Analysis

For Elliptic Fourier analyses (EFA), 40 grape image files were used for each cultivar. EFA analysis was conducted in different phases with the use of MORPHOLOGY (version 1.03) software [36]. In phase I, contours of a closed morphology were defined. In phase II, x and y coordinates of the points on contoured curve were determined. In phase III, coordinate values were converted into mathematical functions. In phase IV, function coefficients were obtained [43]. For function coefficients, analyses were conducted over 20 harmonics. Each harmonic produces four Fourier coefficients (an, bn, cn and dn). The an and bn coefficients correspond to x coordinate and cn and dn coefficients to y coordinate of the curve [44,45].

For image processing, grape images were converted in 24-bit *.bmp format. Four modules were used to obtain morphological data. In module I (ChainCoder), image processing and morphology contour codes were generated. In module II (Chc2Nef), contour codes were normalized and Elliptic Fourier descriptors were obtained. In module III (PrinComp), descriptors were subjected to PC analysis and PC scores were obtained. In module IV (PrinPrint), morphology variations of grape image contours were visualized.

### 2.5. Statistical Assessments

Each variable of morphological characteristics of grape cultivars was subjected to analysis of variance (ANOVA) and significant means were compared with the use of Duncan’s test at 5% significance level. All variables of morphology and dimensional attributes were subjected to Principal Component Analysis (PCA) and differences between the cultivars were presented in scatter plots based on component scores. With PC analysis, significant variables revealing morphology and dimensional differences of the cultivars were identified and ordered. SPSS 20.0 software was used for statistical analyses.

Normalized contour codes by Elliptic Fourier analysis (EFA) were subjected to multivariate variance analysis (MANOVA) with the use of PAST v.4.02 software. Morphological differences between grape cultivars were explained by Hotelling’s pair-wise comparison tests, including verified Bonferroni values and Mahalanobis distances. In linear discriminant analysis conducted with the use of principal component (PC) scores, functions revealing morphology differences of the grape cultivars were determined and similarity relationships were presented in scatter plots. Such similarities were also put forth by hierarchical cluster analysis with the use of Euclidean similarity index and the grape cultivars, with morphology similarities presented in a dendrogram.

## 3. Results and Discussion

### 3.1. Basic Morphology and Dimensional Attributes Measured at Horizontal and Vertical Orientations

Morphology and dimensional attributes of the grape cultivars measured at horizontal and vertical orientations are provided in Table 3. Projection areas measured at both orientations varied in a broad range. Such a case revealed that there were significant physical differences between the grape cultivars. The “Antep karası“, “Hafızali“ and “Royal“ cultivars had the greatest projected areas. Equivalent diameter means were greater at horizontal orientation than at vertical orientation. Increased perimeters were observed in cultivars with the greatest projected area. The greatest elongation average was measured at horizontal orientation. The morphology looks like a full circle as the elongation ratio approaches 1. The lowest elongation ratios were observed in “Yerli beyaz“, “Erenköy beyazı“ and “Amasya beyazı“ cultivars. Previously, Ekhvaia and Akhalkatsi [46] and Leão et al. [47] studied grape genotypes based on berry dimensions and reported high variability. Khadivi-Khub et al. [48] analyzed grape germplasm from Iran based on fruit dimensions and they revealed a significant difference among the evaluated grape cultivars. Kok et al. [49] investigated dimensional attributes of eight grape cultivars in western Turkey and found that berry dimensions were quite variable among eight grape cultivars. Previous studies indicated that grape berry dimensions are cultivar-dependent, yet are affected by numerous factors, including gibberellin treatments, girdling, soil type, irrigation, rootstock and the weather, etc. [50,51,52]. Esgici et al. [53] reported the length, width and thickness of “Şire” grapes, respectively, as 16.16 mm, 15.43 mm and 15.51 mm. Present findings of 20 grape cultivars complied with the values of previous studies.

### 3.2. Basic Morphology and Dimensional Attributes of the Grape Cultivars

The greatest length average was observed in “Antep karası” and the lowest in “Bahçeli karası” cultivar (Table 4). The greatest geometric mean diameter, surface area and volume averages were observed in “Antep karas”, ”Royal” and ”Hafızali” cultivars. In terms of morphology, the grape cultivars with the closest morphology to circle were identified as ”Yerli beyaz”, ”Erenköy beyazı” and ”Amasya beyazı”. Elongation at horizontal orientation designates the longness or shortness of the cultivars. Therefore, the circularity average of 1 measured at horizontal orientation indicates that the morphology is a full circle. The greatest circularity averages were observed in “Bahçeli karası” and “Royal” and the lowest in ”Antep karası” cultivars. Khodaei and Akhijahani [54] reported geometric mean diameters of ”Rasa” grapes based on moisture contents as between 9.20 and 14.74 mm and sphericity values as between 61 and 89%. Esgici et al. [53] reported geometric mean diameter of “Şire” grape as 14.33 mm and sphericity value as 97.1%. In this sense, “Rasa” and “Şire” grape cultivars were similar with “Bahçeli karası” and “Sultani çekirdeksiz (Sultanina)” cultivars.

### 3.3. Eigen Statistics for Two Principal Components

Results of principal component analysis are provided in Table 5. The first two principal components (PC1 and PC2) explained 99.5% of the total variation between the grape cultivars. PC1 had the greatest factor load. The factor loads for dimensional attributes were presented on PC1 and explained 78.5% of the variation between the grape cultivars. In factor-load-based ordering, the dimension variable with the greatest factor load was identified as thickness. It was remarkable that factor loads of dimensional variables (equivalent diameter, projected area and perimeter) measured at vertical orientation were greater than the values measured at horizontal orientation. PC2 explained 20.8% of the variation between the grape cultivars and the greatest factor loads were observed in elongation and sphericity variables. It was remarkable that there was a negative correlation between PC2 and sphericity.

**Figure 4:**
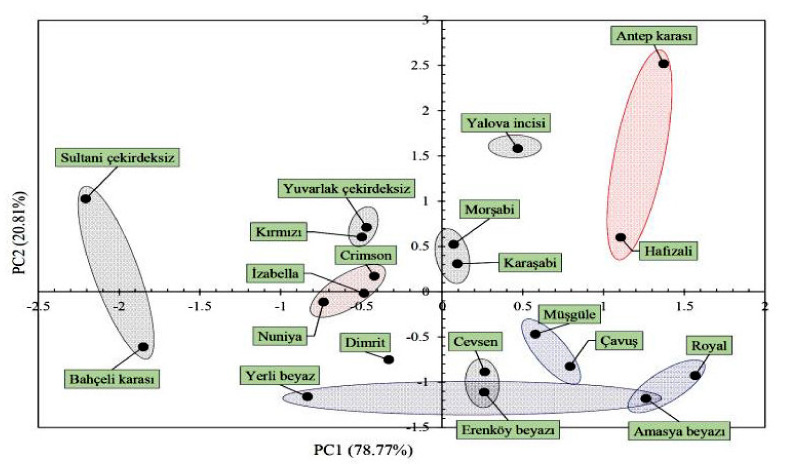
Scatter plot of principal component (PC) loadings defining morphology and dimensional attributes of the grape cultivars.

In the scatter plot presented in Figure 4, dimensional variables had positive correlations with the PC1 axis. The greatest dimensions were seen in “Antep karası“, “Hafızali“, “Royal“ and “Amasya beyazı“ cultivars. On the other hand, “Sultani çekirdeksiz“ and “Bahçeli karası“ cultivars located on the left side of PC1 had the least dimensions. Since sphericity averages had negative correlations with the PC2 axis, the “Antep karası“ cultivar had the least sphericity value. The closest cultivars to each other in terms of both morphology and dimensional attributes were presented in a colored circle. For instance, “Müşgüle“ and “Çavuş“ cultivars had similar morphology and dimensional attributes. “Yuvarlak çekirdeksiz“ and “Kırmızı“ cultivars also had similar morphology and dimensional attributes. The morphology and dimension relationships of the cultivars or genotypes based on morphological characteristics are explained by multivariate statistical analysis methods. Morphological characterization is the first step for the description and classification of grape genotypes and the PCA method is a useful tool for screening the grape genotypes. The PCA method has been used to discriminate grape cultivars by using morphological, biochemical and even molecular data. PCA transforms the original variables into a limited number of uncorrelated new variables. The PCA method also allows the visualization of differences among individuals, the identification of groups and the identification of relationships among individuals and variables [55]. Lamine et al. [56] used the PCA method to discriminate Tunisian grape cultivars and reported high morphological diversity. Nassur et al. [57] used the PCA method on grape cultivars in Brazil, and based on principal component analysis (PCA), all grape cultivars were discriminated and high morphological variation was observed among the accessions. Istrate et al. [58] used applications of the principal component analysis (PCA) at grape varieties from the serogroup Coarnă neagră for establishing phenotypical variability and found great diversity among cultivars. Abiri et al. [55] determined high morphological and pomological variability of a grape (*Vitis vinifera* L.) germplasm collection in Iran by using PCA.

Thusly, in previous studies, differences in cultivars were put forth with principal component analysis in walnuts [42,59,60,61], kiwifruit [62], strawberry tree [63], hazelnuts [33], almonds [35], raspberry [64], citrus [65] and apricot [66]. All above studies indicated that PCA allows the extraction of the maximum information from used cultivars and underlining the interrelations between variables and individuals, either by similarity or opposition.

### 3.4. The Results of the Discriminant Analysis and Pair-Wise Comparisons

**Figure 5:**
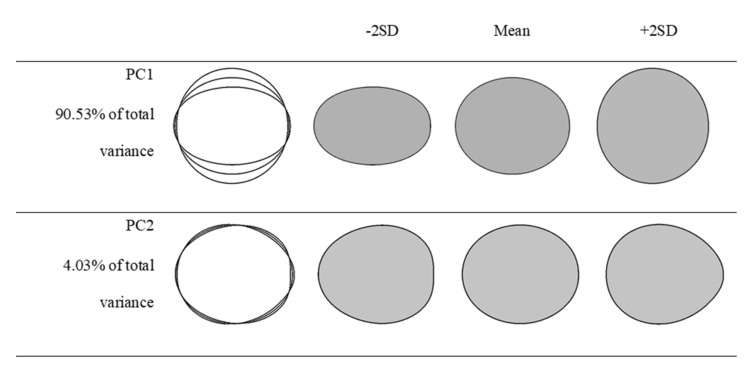
Principal components (PCs) as morphology variables of grape varieties based on a principal component analysis of 800 fruit outlines. From left to right, the outlines show the principal component scores corresponding to: mean −2 standard deviations, mean, mean +2 standard deviations.

The contour codes obtained by Elliptic Fourier Analysis were subjected to principal component analysis (PCA), and morphology differences between the grape cultivars were explained by two principal components (Figure 5). The total variance explained was 94.56%. PC1 explained 90.53% and PC2 explained 4.03% of the total variance. Considering the morphology differences explained by PC1, it was observed that ellipse and sphere geometries constituted the main source of variation. Fruit peduncle or widening at the base of the fruit constituted the source of variation explained by PC2. Grape berry looks similar to a water drop because of this widening. Relative oblateness on fruit surface constituted morphology variations of the genotypes.

According to MANOVA results in Table 6, there were significant contour differences between the grape cultivars. In a linear discriminant analysis conducted with the use of component scores of EFA, two discriminant functions were obtained to discriminate grape contours from each other. The variance explained by the first and second discriminant functions was, respectively, identified as 81.5% and 18.5%. In Hotelling’s pair-wise comparison table, the grape cultivars indicated in color did not have significant morphology differences (*p* > 0.05). If the Mahalanobis distance value provided in pair-wise comparison is low, then the similarity between the cultivars is high. Similar findings were also reported by Demir et al. [38] for *Cornus mas* genotypes.

**Figure 6:**
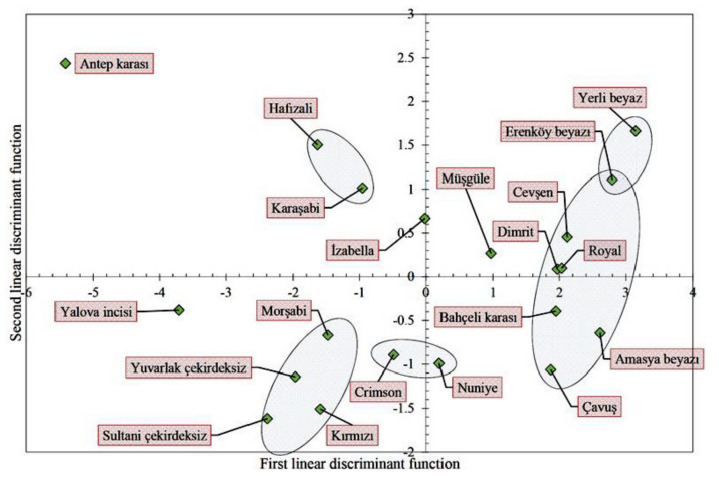
Biplot charts from linear discriminant analysis of 20 grape cultivars based on two principal component morphology variables derived from Elliptic Fourier data of 800 fruit outlines. (The locations of the cultivars on the chart show their own group centroid.)

In Figure 6, the cultivars placed on the right side of the first discriminant function axis look similar to a sphere and the ones on the left side look similar to an ellipse. Peduncle connection section or fruit base is widened as moved away from the axis of the second discriminant function. The cultivars presented in frames in the graph were identified based on the results of the pair-wise comparison test. The cultivars placed in frames had close morphology to each other.

**Figure 7:**
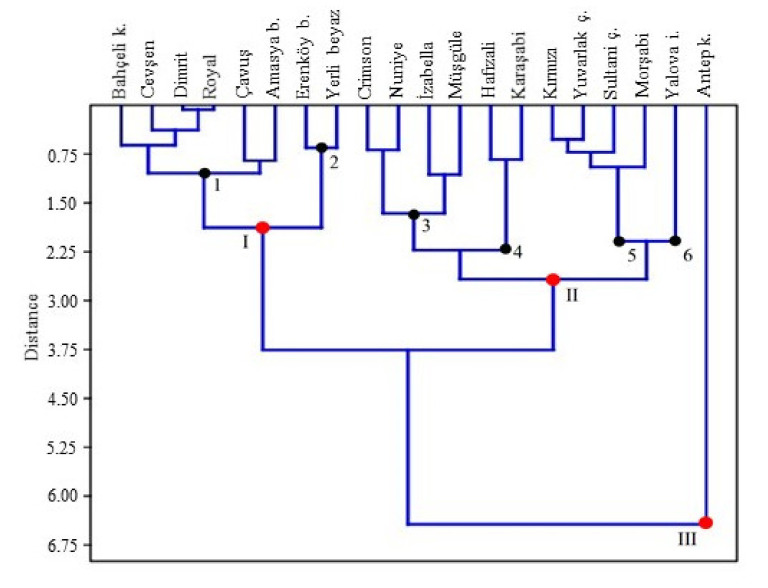
Dendrogram of 20 grape cultivars for the first two principal component scores using hierarchical cluster analysis (between-group linkage method and Euclidean distance).

Results of cluster analysis conducted with the use of group centroids of discriminant functions are provided in Figure 7. Grape cultivars were separated into three morphological groups. Group I and II had six sub-groups. There is only one cultivar in group III.

Figure 7 indicated that the closest cultivars on the dendrogram were Dimrit and Royal, and cultivar Antep karası clearly differed from the rest of the cultivars in terms of berry morphology and dimensions. In fact, as indicated in Figure 1 and Table 4, Antep karası has quite different fruit morphology and diameters than the other cultivars. Thus, group III, including Antep karası, can be classified as out of the group. Previously, morphological data obtained from different grape cultivars showed different clustering patterns on dendrogram, and indicated that grape germplasm in different grapevine growing countries were quite variable in terms of morphological characteristics, which supports to our obtained result [54,55,56,57].

## 4. Conclusions

The projected area, equivalent diameter and perimeter values measured at horizontal orientation were greater than the values measured at vertical orientation. Elongation ratio averages of 20 grape cultivars are not dependent on diameter, area and perimeter of the cultivars. Elongation averages of the cultivars exhibited large variations. The greatest length-to-width ratio was 1.56. There were some cultivars with a morphology quite close to the sphere. When the geometrical dimensions of the cultivars with a high sphericity average were assessed, it was observed that they were both large and small sizes. Principal component analysis revealed that present grape cultivars were distinguished from each other based on dimensional characteristics rather than morphology. Dimensional variables constituted the most significant source of variation between the grape cultivars. The most significant shape variables explaining the variation between the cultivars were identified as sphericity and elongation.

Elliptic Fourier analysis revealed that present grape cultivars looked similar to an ellipse or sphere in morphology. There are also some cultivars that looked similar to a water drop because of widening at the peduncle or fruit base. Linear discriminant analysis and cluster analysis clearly demonstrated similarities between the grape cultivars. In terms of morphology, grape cultivars were classified into 7 sub-groups. The first sub-group included “Bahçeli karası“, “Cevşen“, “Dimrit“, “Royal“, “Çavuş“ and “Amasya beyazı“ cultivars; the second group included “Erenköy beyazı“ and “Yerli beyaz“ cultivars; the third group included “Crimson“, “Nuniya“, “İzabella“ and “Müşgüle“ cultivars; the fourth sub-group included “Hafızali“ and “Karaşabi“ cultivars; the fifth sub-group included “Kırmızı“, “Yuvarlak çekirdeksiz“, “Sultani çekirdeksiz“ and “Morşabi“ cultivars; the sixth sub-group included “Yalova incisi“ and the seventh sub-group included the “Antep karası“ cultivar.

## Figures and Tables

**Table 1:** External (skin) berry color of cultivars.

Cultivars	Berry Skin Color
*Amasya beyazı*	Green-yellow
Antep karası	Black
Bahçeli karası	Black
Çavuş	Green-yellow
Cevşen	Green-yellow
Crimson	Dark red
Dimrit	Purple-black
Erenköy beyazı	Green-yellow
Hafizali	Green-yellow
Karaşabi	Black
Kırmızı	Dark red
Izabella (İsabella)	Dark purple-black
Morşabi	Purple
Müşgüle	Green-yellow
Nuniya	Green-yellow
Royal	Black
Sultani çekirdeksiz (Sultanina)	Green-yellow
Yalova incisi	Green-yellow
Yerli beyaz	Green-yellow
Yuvarlak çekirdeksiz	Green-ellow

**Table 2:** Equations used for morphology and dimensional attributes of the grape cultivars.

Morphological–Dimensional Attributes	Equations	References
Aspect ratio (AR)	$AR_{h}=L/W$; for hor. orientation $AR_{v}=W/T$; for vertical orientation	[38]
Geometric mean diameter (*D_g_*, mm)	$D_{g}=\sqrt[{3}]{\left(L\cdot W\cdot T\right)}$	[39]
Sphericity (*φ,* %)	$\varphi =\left(D_{g}/L\right)\cdot 100$	[40]
Volume (*V*, mm^3^)	$V=\left(\pi /6\right)\cdot \left(L\cdot W\cdot T\right)$	Ellipse volume
Surface area (*SA*, mm^2^)	$SA=\pi \cdot D_{g}^{2}$	[41]
Circularity (*C*)	$C=4\cdot \pi \cdot PA/P^{2}$	[42]

**Table 3:** Basic morphology and dimensional attributes measured at horizontal and vertical orientations.

Varieties	Horizontal Orientation				Vertical Orientation			
	Projected Area (mm^2^)	Feret Diameter (mm)	Perimeter (mm)	Aspect Ratio	Projected Area (mm^2^)	Feret Diameter (mm)	Perimeter (mm)	Aspect Ratio
Amasya beyazı	371.2 ± 28.0 ^d,^*	21.73±0.81 ^d^	73.68 ± 2.94 ^d^	1.072 ± 0.042 ^klm^	356.8 ± 31.0 ^b^	21.30 ± 0.91 ^b^	71.31 ± 3.11 ^b^	1.028 ± 0.016 ^f^
Antep karası	516.1 ± 47.9 ^a^	25.61 ± 1.19 ^a^	89.21 ± 4.21 ^a^	1.557 ± 0.103 ^a^	347.6 ± 37.6 ^bc^	21.01 ± 1.14 ^bc^	70.52 ± 4.01 ^b^	1.057 ± 0.041 ^a^
Bahçeli karası	150.8 ± 14.2 ^m^	13.84 ± 0.65 ^o^	46.51 ± 2.26 ^o^	1.100 ± 0.035 ^j^	136.4 ± 13.7 ^j^	13.16 ± 0.65 ^j^	44.73 ± 2.30 ^j^	1.032 ± 0.017 ^cdef^
Çavuş	345.3 ± 37.6 ^e^	20.94 ± 1.13 ^e^	71.34 ± 4.15 ^e^	1.103 ± 0.044 ^j^	316.0 ± 40.4 ^d^	20.02 ± 1.27 ^d^	68.00 ± 4.06 ^c^	1.036 ± 0.018 ^bcdef^
Cevşen	297.6 ± 16.2 ^h^	19.46 ± 0.53 ^h^	65.97 ± 1.83 ^h^	1.095 ± 0.039 ^jkl^	278.3 ± 17.3 ^f^	18.81 ± 0.58 ^f^	63.32 ± 1.99 ^e^	1.034 ± 0.017 ^cdef^
Crimson	276.4 ± 29.3 ^i^	18.73 ± 0.98 ^j^	63.63 ± 3.38 ^ji^	1.218 ± 0.056 ^g^	229.1 ± 22.4 ^h^	17.06 ± 0.83 ^h^	57.16 ± 2.71 ^h^	1.032 ± 0.017 ^cdef^
Dimrit	256.6 ± 18.2 ^j^	18.06 ± 0.64 ^k^	61.27 ± 2.10 ^k^	1.100 ± 0.042 ^j^	233.7 ± 17.5 ^h^	17.24 ± 0.64 ^h^	58.52 ± 2.07 ^g^	1.036 ± 0.018 ^bcdef^
Erenköy beyazı	289.9 ± 18.9 ^hi^	19.20 ± 0.63 ^hi^	65.40 ± 2.42 ^hi^	1.069 ± 0.028 ^lm^	277.6 ± 20.3 ^f^	18.79 ± 0.68 ^f^	63.55 ± 2.44 ^e^	1.027 ± 0.016 ^f^
Hafizali	422.8 ± 37.6 ^b^	23.18 ± 1.04 ^b^	79.03 ± 3.88 ^b^	1.285 ± 0.058 ^de^	337.1 ± 30.3 ^c^	20.70 ± 0.93 ^c^	69.14 ± 3.09 ^c^	1.031 ± 0.017 ^cdef^
Karaşabi	320.1 ± 27.4 ^g^	20.17 ± 0.87 ^g^	68.22 ± 3.08 ^g^	1.245 ± 0.059 ^f^	265.5 ± 24.2 ^g^	18.37 ± 0.85 ^g^	61.40 ± 2.82 ^f^	1.042 ± 0.027 ^bcd^
Kırmızı	282.7 ± 25.3 ^i^	18.95 ± 0.83 ^ij^	64.40 ± 2.96 ^ji^	1.277 ± 0.061 ^de^	223.1 ± 19.9 ^h^	16.84 ± 0.74 ^h^	56.47 ± 2.35 ^h^	1.031 ± 0.020 ^def^
İzabella	262.6 ± 27.5 ^j^	18.26 ± 0.95 ^k^	61.92 ± 3.27 ^k^	1.198 ± 0.058 ^hg^	225.7 ± 27.0 ^h^	16.92 ± 1.00 ^h^	56.73 ± 3.34 ^h^	1.036 ± 0.024 ^bcdef^
Morşabi	327.5 ± 29.0 ^fg^	20.40 ± 0.91 ^fg^	69.44 ± 3.29 ^fg^	1.266 ± 0.057 ^ef^	262.3 ± 25.6 ^g^	18.25 ± 0.90 ^g^	61.22 ± 2.97 ^f^	1.030 ± 0.016 ^ef^
Müşgüle	338.6 ± 22.6 ^ef^	20.75 ± 0.69 ^ef^	70.65 ± 2.43 ^ef^	1.146 ± 0.052 ^i^	299.8 ± 18.6 ^e^	19.53 ± 0.60 ^e^	66.00 ± 1.86 ^d^	1.034 ± 0.016 ^cdef^
Nuniya	242.5 ± 25.8 ^k^	17.55 ± 0.94 ^ij^	59.31 ± 3.19 ^l^	1.180 ± 0.050 ^h^	208.1 ± 23.8 ^i^	16.25 ± 0.93 ^i^	54.56 ± 3.12 ^i^	1.040 ± 0.026 ^bcde^
Royal	410.5 ± 43.0 ^bc^	22.83 ± 1.19 ^bc^	76.80 ± 3.96 ^c^	1.097 ± 0.055 ^ij^	379.7 ± 43.3 ^a^	21.95 ± 1.23 ^a^	73.26 ± 4.06 ^a^	1.046 ± 0.020 ^b^
Sultani çekirdeksiz	159.7 ± 9.8 m	14.25 ± 0.44 n	48.99 ± 1.72 n	1.326 ± 0.070 c	120.6 ± 8.4 k	12.38 ± 0.43 k	42.12 ± 1.67 k	1.042 ± 0.025 bc
Yalova incisi	401.1 ± 50.2 ^c^	22.56 ± 1.40 ^c^	77.63 ± 4.90 ^c^	1.415 ± 0.080 ^b^	285.4 ± 40.5 ^f^	19.02 ± 1.34 ^f^	65.51 ± 4.99 ^d^	1.034 ± 0.017 ^cdef^
Yerli beyaz	201.3 ± 17.2 ^l^	16.00 ± 0.68 ^m^	55.09 ± 2.53 ^m^	1.055 ± 0.029 ^m^	201.9 ± 15.6 ^i^	16.02 ± 0.62 ^i^	54.26 ± 2.10 ^i^	1.033 ± 0.018 ^cdef^
Yuvarlak çekirdeksiz	286.2 ± 20.8 ^hi^	19.08 ± 0.69 ^hij^	65.17 ± 2.49 ^hi^	1.297 ± 0.056 ^d^	225.4 ± 16.6 ^h^	16.93 ± 0.62 ^h^	56.86 ± 2.02 ^h^	1.037 ± 0.018 ^bcdef^
Mean ± SD	308.0 ± 92.0	19.58 ± 2.98	66.68 ± 10.38	1.205 ± 0.140	260.5 ± 71.9	18.03 ± 2.59	60.73 ± 8.60	1.036 ± 0.022
Min-max	119.8–619.4	12.35–28.08	41.41–98.43	1.008–1.782	106.9–518.4	11.67–25.69	39.2–85.49	1.000–1.216

*: Means followed by the same letter in the same column are not significantly different based on Duncan’s test at 5% significance level.

**Table 4:** Basic morphology and dimensional attributes of the grape cultivars.

Varieties	Length (mm)	Width (mm)	Thickness (mm)	Geometric Mean Diameter (mm)	Sphericity (%)	Surface Area (cm^2^)	Volume (cm^3^)	Circularity
Amasya beyazı	22.67 ± 0.92 ^de*^	21.17 ± 0.96 ^b^	21.46 ± 0.93 ^b^	21.75±0.84 ^c^	96.0 ± 2.0 ^a^	14.89 ± 1.16 ^c^	5.413 ± 0.638 ^c^	0.858 ± 0.020 ^bcd^
Antep karası	31.94 ± 1.69 ^a^	20.56 ± 1.22 ^c^	21.52 ± 1.42 ^b^	24.17 ± 1.20 ^a^	75.7 ± 3.1 ^i^	18.39 ± 1.82 ^a^	7.443 ± 1.104 ^a^	0.813 ± 0.017 ^h^
Bahçeli karası	14.54 ± 0.77 ^l^	13.22 ± 0.67 ^k^	13.20 ± 0.72 ^k^	13.64 ± 0.67 ^m^	93.8 ± 1.9 ^b^	5.86 ± 0.58 ^l^	1.338 ± 0.202 ^l^	0.874 ± 0.012 ^a^
Çavuş	22.14 ± 1.17 ^ef^	20.10 ± 1.24 ^d^	20.11 ± 1.47 ^d^	20.76 ± 1.22 ^e^	93.7 ± 2.4 ^b^	13.58 ± 1.60 ^e^	4.731 ± 0.844 ^e^	0.851 ± 0.020 ^def^
Cevşen	20.54 ± 0.69 ^hi^	18.78 ± 0.56 ^f^	18.93 ± 0.72 ^fg^	19.40 ± 0.53 ^fg^	94.5 ± 2.2 ^b^	11.83 ± 0.64 ^fg^	3.828 ± 0.311 ^fg^	0.859 ± 0.016 ^bcd^
Crimson	20.73 ± 1.27 ^h^	17.02 ± 0.84 ^hi^	17.16 ± 0.92 ^i^	18.22 ± 0.90 ^hij^	88.0 ± 2.4 ^e^	10.45 ± 1.04 ^hi^	3.189 ± 0.479 ^hi^	0.856 ± 0.013 ^cde^
Dimrit	19.06 ± 0.74 ^j^	17.34 ± 0.72 ^hi^	17.28 ± 0.69 ^i^	17.87 ± 0.61 ^j^	93.8 ± 2.1 ^b^	10.04 ± 0.68 ^i^	2.997 ± 0.307 ^i^	0.858 ± 0.014 ^bcd^
Erenköy beyazı	20.03 ± 0.72 ^i^	18.75 ± 0.71 ^f^	18.93 ± 0.75 ^fg^	19.23 ± 0.67 ^g^	96.0 ± 1.5 ^a^	11.63 ± 0.82 ^g^	3.736 ± 0.396 ^g^	0.851 ± 0.016 ^def^
Hafizali	26.36 ± 1.47 ^b^	20.53 ± 0.95 ^c^	20.92 ± 1.00 ^c^	22.45 ± 1.00 ^b^	85.3 ± 2.4 ^f^	15.86 ± 1.41 ^b^	5.957 ± 0.790 ^b^	0.849 ± 0.015 ^ef^
Karaşabi	22.68 ± 1.22 ^de^	18.23 ± 0.87 ^g^	18.59 ± 0.99 ^gh^	19.73 ± 0.88 ^f^	87.1 ± 2.7 ^e^	12.25 ± 1.08 ^f^	4.043 ± 0.530 ^f^	0.863 ± 0.011 ^bc^
Kırmızı	21.42 ± 1.17 ^g^	16.79 ± 0.77 ^i^	17.03 ± 0.84 ^i^	18.29 ± 0.81 ^hi^	85.5 ± 2.5 ^f^	10.53 ± 0.95 ^hi^	3.224 ± 0.447 ^hi^	0.855 ± 0.013 ^cde^
İzabella	20.13 ± 1.14 ^i^	16.82 ± 1.03 ^i^	17.12 ± 1.09 ^i^	17.96 ± 0.99 ^ij^	89.3 ± 2.7 ^d^	10.16 ± 1.13 ^hi^	3.059 ± 0.518 ^hi^	0.859 ± 0.017 ^bcd^
Morşabi	23.08 ± 1.18 ^d^	18.24 ± 0.9 ^g^	18.35 ± 0.97 ^h^	19.76 ± 0.90 ^f^	85.7 ± 2.4 ^f^	12.29 ± 1.12 ^f^	4.065 ± 0.552 ^f^	0.852 ± 0.013 ^def^
Müşgüle	22.32 ± 0.99 ^e^	19.49 ± 0.68 ^e^	19.65 ± 0.74 ^e^	20.44 ± 0.66 ^e^	91.6 ± 2.3 ^c^	13.14 ± 0.85 ^e^	4.485 ± 0.440 ^e^	0.852 ± 0.019 ^def^
Nuniya	19.13 ± 1.07 ^j^	16.24 ± 1.00 ^j^	16.37 ± 0.95 ^j^	17.19 ± 0.90 ^k^	89.9 ± 2.5 ^d^	9.31 ± 0.97 ^j^	2.681 ± 0.418 ^j^	0.864 ± 0.010 ^b^
Royal	24.10 ± 1.37 ^c^	21.99 ± 1.30 ^a^	21.99 ± 1.34 ^a^	22.66 ± 1.19 ^b^	94.1 ± 2.8 ^b^	16.18 ± 1.72 ^b^	6.145 ± 0.991 ^b^	0.872 ± 0.012 ^a^
Sultani çekirdeksiz	16.37 ± 0.75 ^k^	12.36 ± 0.45 ^l^	12.53 ± 0.54 ^l^	13.63 ± 0.42 ^m^	83.4 ± 3.0 ^g^	5.84 ± 0.36 ^l^	1.330 ± 0.125 ^l^	0.836 ± 0.020 ^g^
Yalova incisi	26.69 ± 1.76 ^b^	18.90 ± 1.31 ^f^	19.20 ± 1.41 ^f^	21.31 ± 1.35 ^d^	79.9 ± 2.9 ^h^	14.32 ± 1.84 ^d^	5.125 ± 0.994 ^d^	0.833 ± 0.012 ^g^
Yerli beyaz	16.71 ± 0.80 ^k^	15.84 ± 0.67 ^j^	16.12 ± 0.70 ^j^	16.22 ± 0.67 ^l^	97.1 ± 1.5 ^a^	8.27 ± 0.69 ^k^	2.244 ± 0.278 ^k^	0.833 ± 0.028 ^g^
Yuvarlak çekirdeksiz	21.72 ± 1.04 ^fg^	16.75 ± 0.64 ^i^	17.24 ± 0.72 ^i^	18.44 ± 0.67 ^h^	85.0 ± 2.5 ^f^	10.69 ± 0.78 ^h^	3.294 ± 0.36 ^h^	0.846 ± 0.012 ^f^
Mean ± SD	21.62 ± 3.96	17.96 ± 2.57	18.19 ± 2.65	19.16 ± 2.82	89.3 ± 6.2	11.78 ± 3.36	3.916 ± 1.644	0.852 ± 0.021
Min-max	12.53–35.78	11.49–25.54	11.53–25.91	12.37–26.44	71.1–99.5	4.81–21.96	0.992–9.676	0.769–0.890

*: Means followed by the same letter in the same column are not significantly different based on Duncan’s test at 5% significance level.

**Table 5:** Eigen statistics for two principal components.

Variables	PC1	PC2
Thickness	**0.999**	−0.006
Feret diameter at vertical orientation.	**0.998**	−0.039
Perimeter at vertical orientation.	**0.998**	−0.036
Projected area at vertical orientation.	**0.997**	−0.032
Width	**0.996**	−0.069
Geometric mean diameter	**0.976**	0.212
Surface area	**0.968**	0.247
Volume	**0.951**	0.280
Feret diameter at horizontal orientation.	**0.940**	0.337
Perimeter at horizontal orientation.	**0.927**	0.372
Projected area at horizontal orientation.	**0.923**	0.380
Length	**0.811**	0.583
Aspect ratio at horizontal orientation.	0.075	**0.996**
Sphericity	**−0.029**	−0.996
Eigenvalues	11.028	2.914
% of variance	78.773	20.812
Cumulative (%)	78.773	99.585

**Table 6:** The results of the discriminant analysis and pair-wise comparisons.

A. Canonical Discriminant Functions (35.9% of Original Grouped Cases Correctly Classified) (Computed in SPSS ver. 20)																													
**Functions**				**Eigenvalue**					**% of Variance**								**Cumulative %**						**Canonical Correlation**						
**1**				**5.461**					**81.5**								**81.5**						**0.919**						
**2**				**1.241**					**18.5**								**100.0**						**0.744**						
**B. MANOVA Results (Computed in PAST ver. 4.05)**																													
**Statistics**			**Value**					**Hypothesis df**						**Error df**						**F Value**						***p* (Sigma)**			
Wilks’ lambda			0.06904					38						1558						115.00						0.000			
Pillai trace			1.399					38						1560						95.59						0.000			
C. Hotelling’s Pair-Wise Comparisons. (Bonferroni Corrected *p* Values in Upper Triangle; Mahalonabis Distances in Lower Triangle) (Computed in PAST ver. 4.05) *																													
Cultivars	Amasya beyazı	Antep karası			Bahçeli karası	Cevşen	Crimson			Çavuş	Dimrit	Erenköy beyazı	Hafızali		İzabella	Karaşabi		Kırmızı	Morşabi		Müşgüle	Nuniye		Royal	Sultani çekirdeksiz		Yalova incisi	Yerli beyaz	Yuvarlak çekirdeksiz
Amasya beyazı		1.6E−48b *			1.8E+00	9.9E−04	2.8E−19			2.7E−01	4.4E−02	3.3E−08	1.7E−30		5.9E−18	3.1E−25		1.3E−27	2.8E−26		3.3E−09	5.8E−14		7.4E−02	1.7E−32		6.3E−39	2.5E−13	1.4E−29
Antep karası	73.99				8.7E−46	2.1E−45	4.4E−37			1.5E−46	3.1E−45	1.8E−47	5.0E−25		1.0E−35	4.4E−30		1.0E−34	4.8E−32		6.5E−41	4.0E−40		1.8E−45	2.7E−32		1.0E−20	1.7E−48	8.9E−32
Bahçeli karası	0.50	62.27				2.2E−01	2.7E−14			2.8E+00	2.1E+01	6.4E−08	4.9E−26		3.3E−12	3.6E−20		1.1E−23	1.0E−21		1.5E−03	5.5E−09		1.7E+01	5.7E−29		1.5E−35	2.0E−13	1.4E−25
Cevşen	1.45	60.75			0.75		6.6E−18			2.3E−06	4.1E+01	7.3E−02	5.6E−25		1.7E−11	2.1E−19		5.4E−27	4.3E−24		2.0E−03	1.1E−13		5.2E+01	1.0E−31		9.5E−37	9.5E−07	3.2E−28
Crimson	9.65	35.42			6.16	8.59				2.7E−13	1.3E−15	1.3E−24	1.0E−15		4.1E−07	6.3E−10		3.3E−04	2.3E−02		4.5E−09	2.3E+00		3.9E−16	1.4E−10		1.9E−20	1.5E−28	4.4E−06
Çavuş	0.72	65.38			0.45	2.36	5.58				2.4E−03	2.9E−13	6.3E−28		6.3E−15	3.0E−22		3.9E−22	3.0E−21		6.3E−07	1.6E−07		1.7E−03	1.2E−27		2.3E−35	1.7E−18	1.4E−24
Dimrit	0.95	60.10			0.23	0.16	6.98			1.33		1.4E−04	8.4E−25		7.9E−11	5.8E−19		5.1E−25	2.1E−22		2.3E−02	6.9E−11		1.8E+02	4.9E−30		9.2E−36	6.0E−10	1.6E−26
Erenköy beyazı	3.09	69.28			2.97	0.88	14.76			5.56	1.72		1.3E−28		3.0E−17	5.3E−24		1.5E−32	1.0E−29		2.5E−10	4.8E−21		3.5E−04	1.3E−36		1.5E−40	3.4E+00	1.9E−33
Hafızali	22.61	15.22			16.40	15.16	7.05			18.84	14.96	19.77			1.0E−08	3.4E−01		1.5E−18	9.4E−12		1.6E−17	3.7E−19		3.3E−25	4.5E−20		6.6E−17	1.3E−30	8.5E−16
İzabella	8.63	32.32			4.98	4.61	2.65			6.55	4.28	8.11	3.31			2.9E−02		6.0E−16	4.6E−10		1.0E−02	1.7E−07		2.4E−11	1.1E−20		1.4E−24	7.8E−21	9.6E−16
Karaşabi	15.46	21.96			10.40	9.75	3.85			12.29	9.40	14.08	0.70		1.00			2.9E−15	3.1E−08		8.4E−11	7.1E−13		2.0E−19	2.0E−18		4.1E−19	1.1E−26	1.9E−13
Kırmızı	18.41	30.22			13.75	17.61	1.60			12.18	15.21	26.08	9.07		7.21	6.76			2.9E−01		2.4E−19	4.7E−09		1.8E−25	5.0E−01		1.4E−13	9.0E−36	1.4E+01
Morşabi	16.71	25.20			11.79	14.18	1.03			11.35	12.44	21.41	4.74		3.91	3.10		0.72			2.0E−15	9.9E−08		7.0E−23	1.3E−04		2.3E−12	6.3E−33	2.5E+00
Müşgüle	3.52	45.55			1.39	1.35	3.46			2.58	1.03	4.04	8.30		1.14	4.26		9.72	6.87			6.8E−06		8.3E−03	1.1E−24		2.7E−30	4.2E−15	2.0E−20
Nuniye	5.96	43.27			3.43	5.79	0.47			2.81	4.31	11.17	9.56		2.80	5.34		3.45	2.90		2.19			2.0E−11	9.6E−16		2.4E−25	1.8E−25	1.2E−11
Royal	0.88	61.05			0.25	0.13	7.33			1.37	0.00	1.60	15.42		4.54	9.78		15.73	12.92		1.16	4.57			1.9E−30		4.1E−36	1.5E−09	5.9E−27
Sultani çekirdeksiz	25.97	25.61			20.30	24.62	4.16			18.46	21.90	34.32	10.32		10.85	8.98		0.65	1.74		14.85	7.07		22.52			1.3E−08	1.9E−39	4.2E+00
Yalova incisi	40.00	10.88			31.97	34.64	10.64			31.57	32.43	44.52	7.86		14.72	9.53		5.75	5.06		22.31	15.60		33.21	3.26			1.1E−42	2.1E−09
Yerli beyaz	5.59	73.92			5.66	2.51	19.68			9.04	3.86	0.43	22.82		10.98	17.21		32.46	26.77		6.66	15.73		3.67	41.37		51.10		1.9E−36
Yuvarlak çekirdeksiz	21.21	24.72			15.88	19.24	2.26			14.73	17.00	27.74	7.11		7.07	5.67		0.28	0.46		10.62	4.70		17.55	0.41		3.61	33.97	

*: The grape varieties shown in color are not significantly different in terms of shape (*p* > 0.05 insignificant).

## Data Availability

All-new research data were presented in this contribution.
